# Supplementary figures and images for: An indigenous Saccharomyces uvarum population with high genetic diversity dominates uninoculated Chardonnay fermentations at a Canadian winery
Source: PLoS One. 2021 Feb 4;16(2):e0225615. doi: 10.1371/journal.pone.0225615 (PMC7861373; doi:10.1371/journal.pone.0225615)

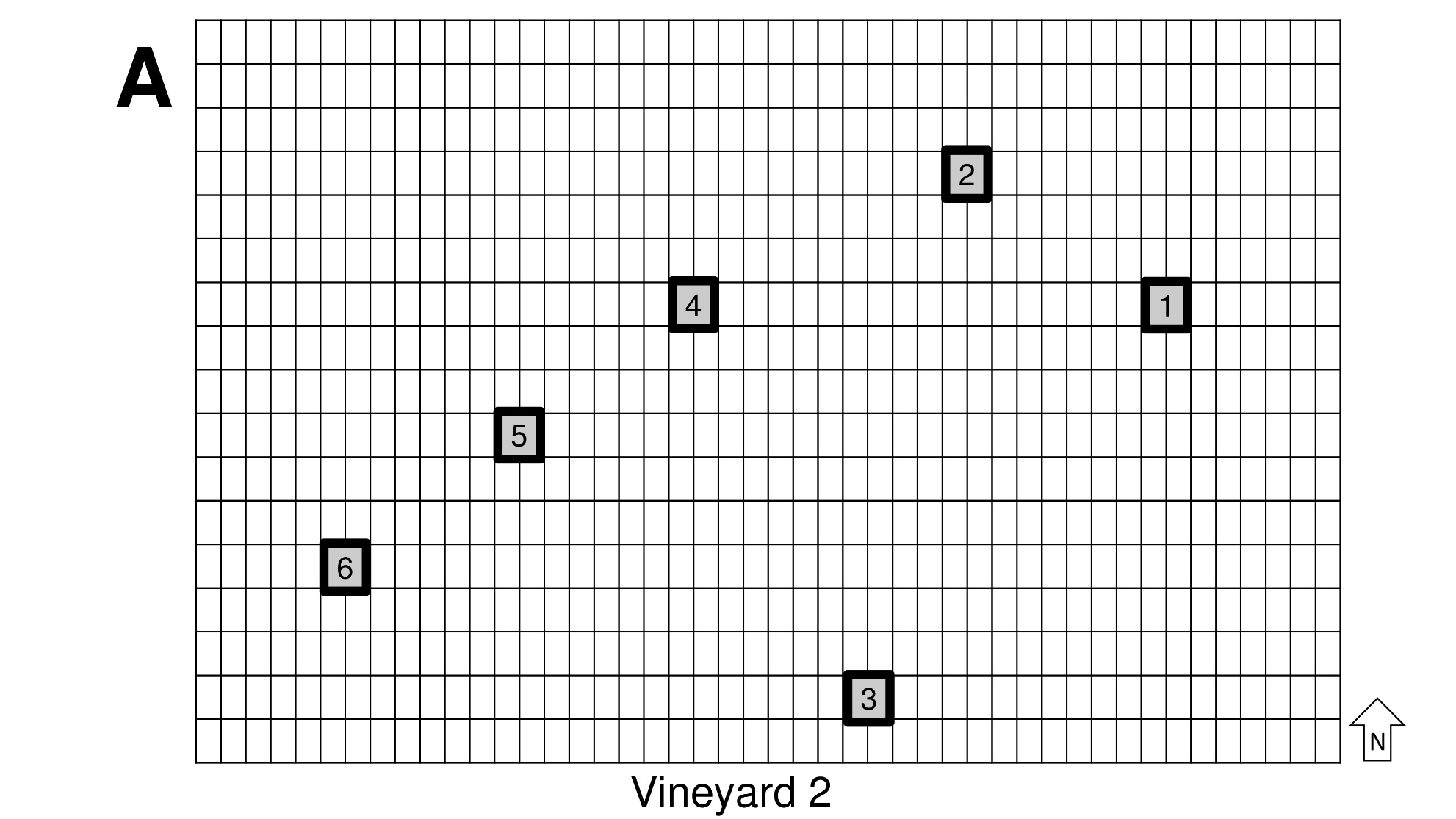


**Fig S1A.**


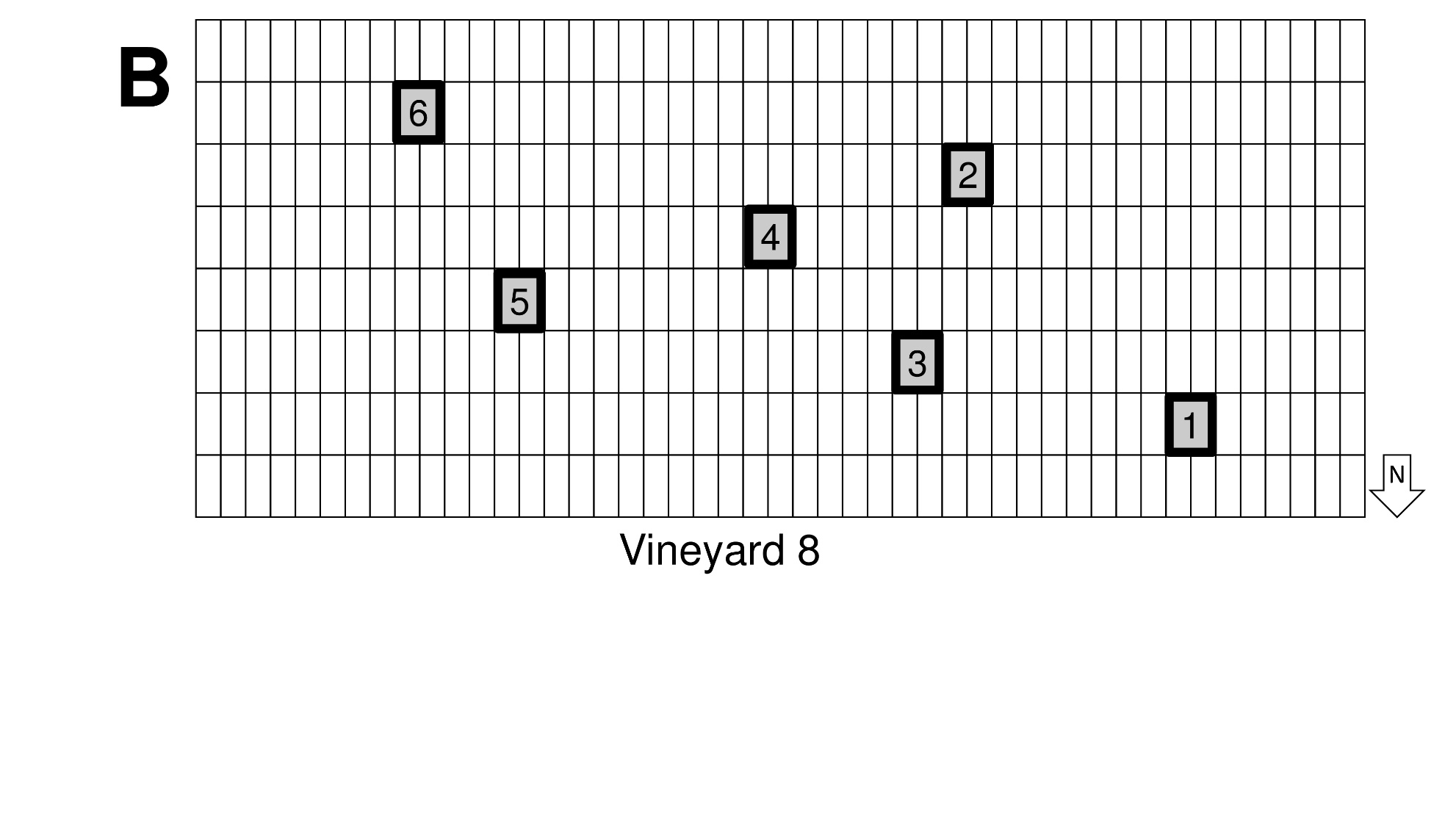


**Fig S1B.**

Supplement: S1 Fig — Sampling layouts for (A) Vineyard 2 and (B) Vineyard 8. Each of the two conjoined squares represent the generated site of collection for one sample, with the sample number also given at each sampling site. One sampling site contains approximately 15 vines, and two grape clusters were taken from each vine (one on either side of the row), for a total of 30 clusters per sampling site. The geographic orientation of each vineyard is indicated in the bottom right corner of each sampling map. (DOCX) [file pone.0225615.s001.docx]

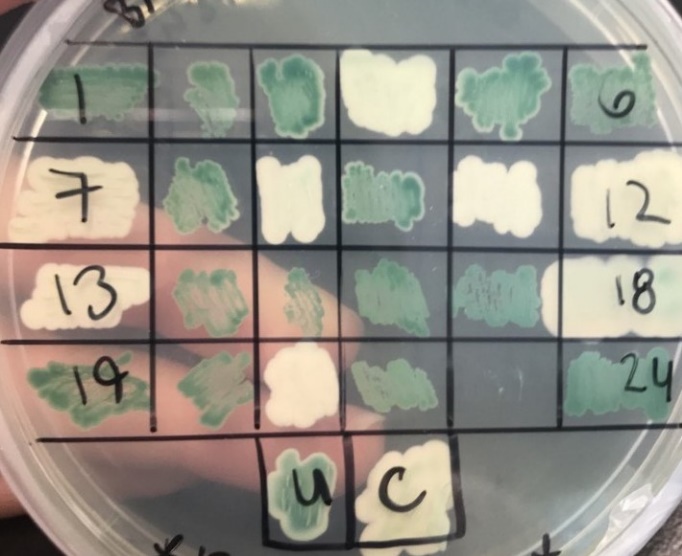


**Fig S2A.**


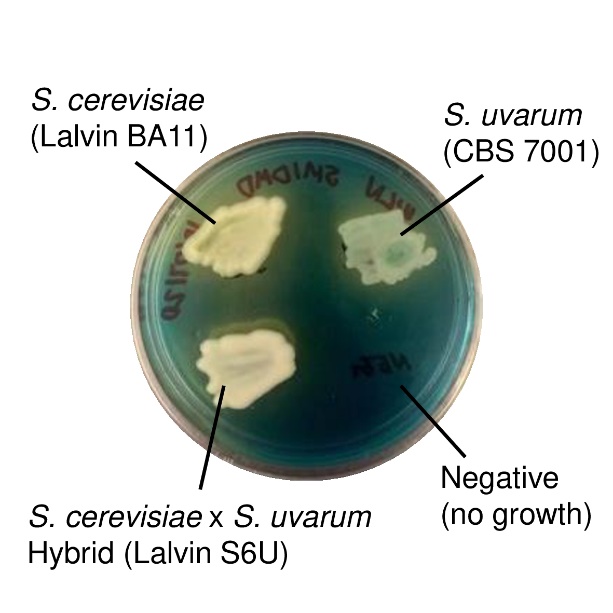


**Fig S2B.**

Supplement: S2 Fig — (A) Forty-seven yeast colonies per barrel, per sampling stage, were isolated and plated onto WLN media in order to distinguish between presumed S. cerevisiae and presumed S. uvarum isolates prior to strain-typing. On each plate, an S. cerevisiae control (Lalvin BA11) and an S. uvarum control (CBS 7001) were used to help aid the differentiation between the two species. Presumed S. cerevisiae isolates appeared cream-coloured, while presumed S. uvarum isolates appeared green. (B) Comparison of colony colour of a pure S. cerevisiae strain (Lalvin BA11), a pure S. uvarum strain (CBS 7001), and a S. cerevisiae x S. uvarum hybrid (Lalvin S6U), plated on WLN media. (DOCX) [file pone.0225615.s002.docx]

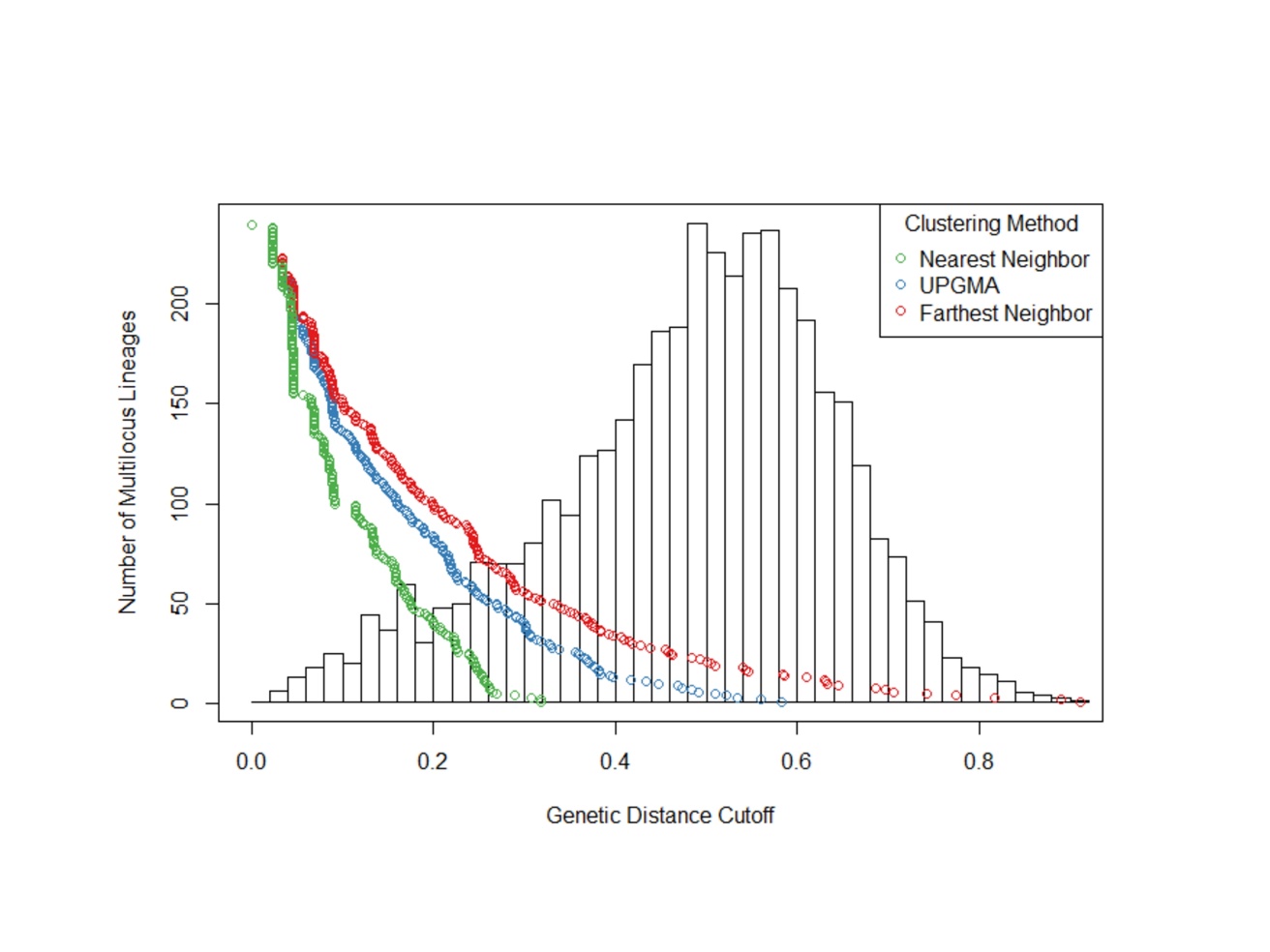


**Fig S3.**

Supplement: S3 Fig — The plot shows genetic distance cutoff as a function of the number of multilocus lineages, depending on the clustering method used. (DOCX) [file pone.0225615.s003.docx]

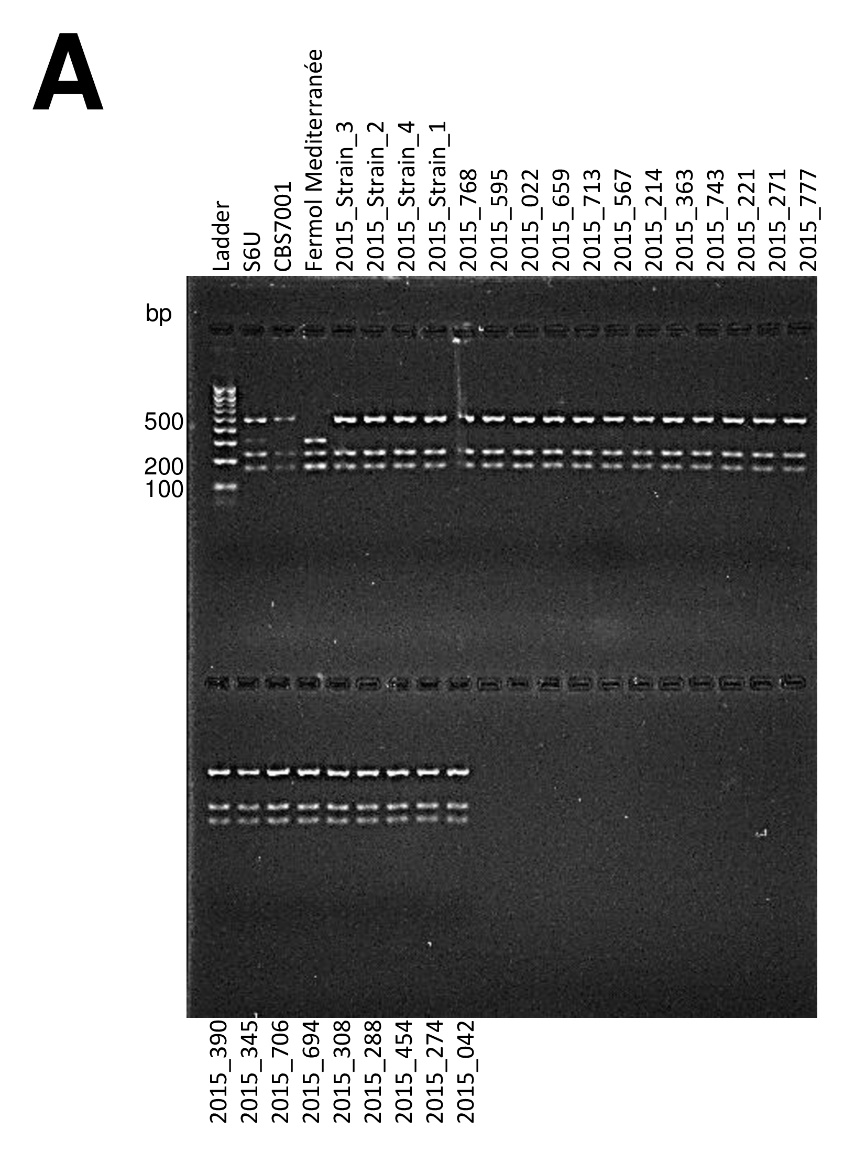


**Fig S4A.**


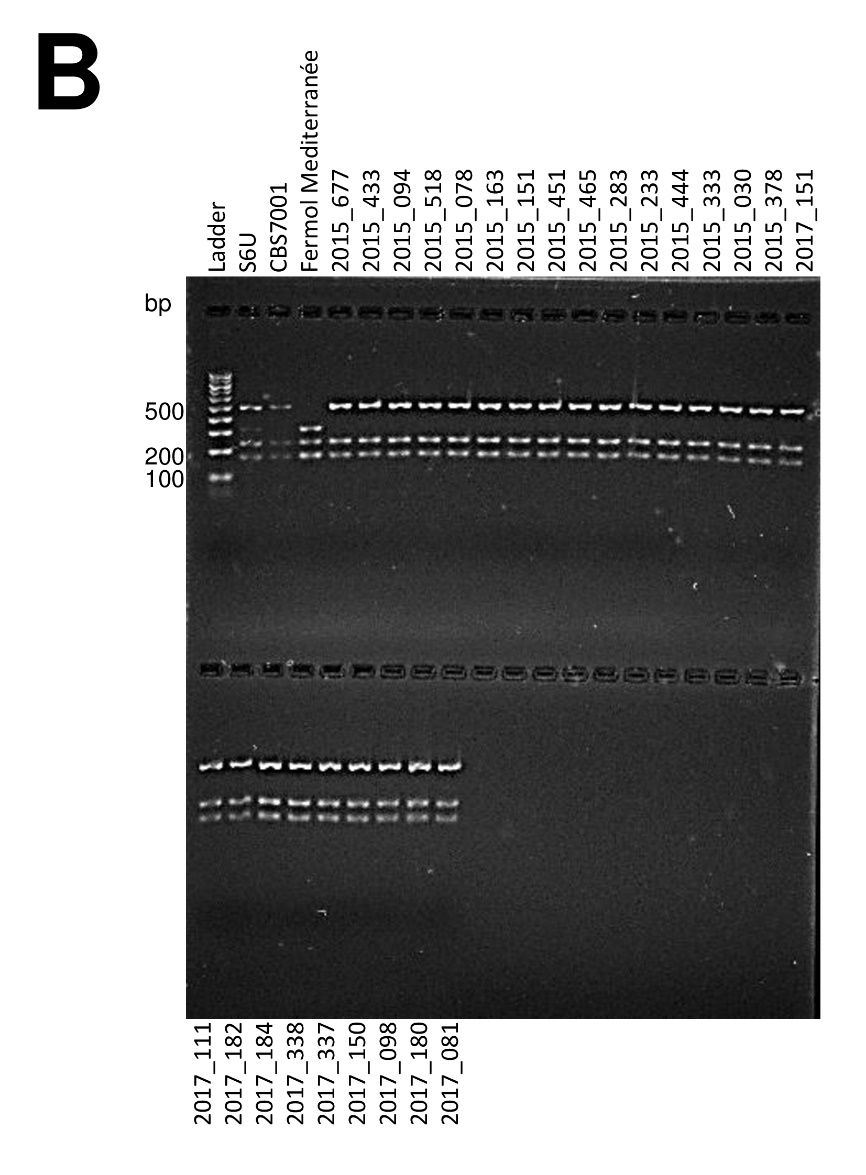


**Fig S4B.**

Supplement: S4 Fig — Restriction digest profiles of 50 yeast isolates, representing the 50 most abundant strains found in this study, as compared to reference strains: a pure S. cerevisiae strain (Fermol Mediterranée), a pure S. uvarum strain (CBS 7001), and a S. cerevisiae x S. uvarum hybrid strain (Lalvin S6U). (A) First 25 strains, as well as a ladder and all three reference strains. (B) Second 25 strains, as well as a ladder and all three reference strains. (DOCX) [file pone.0225615.s004.docx]

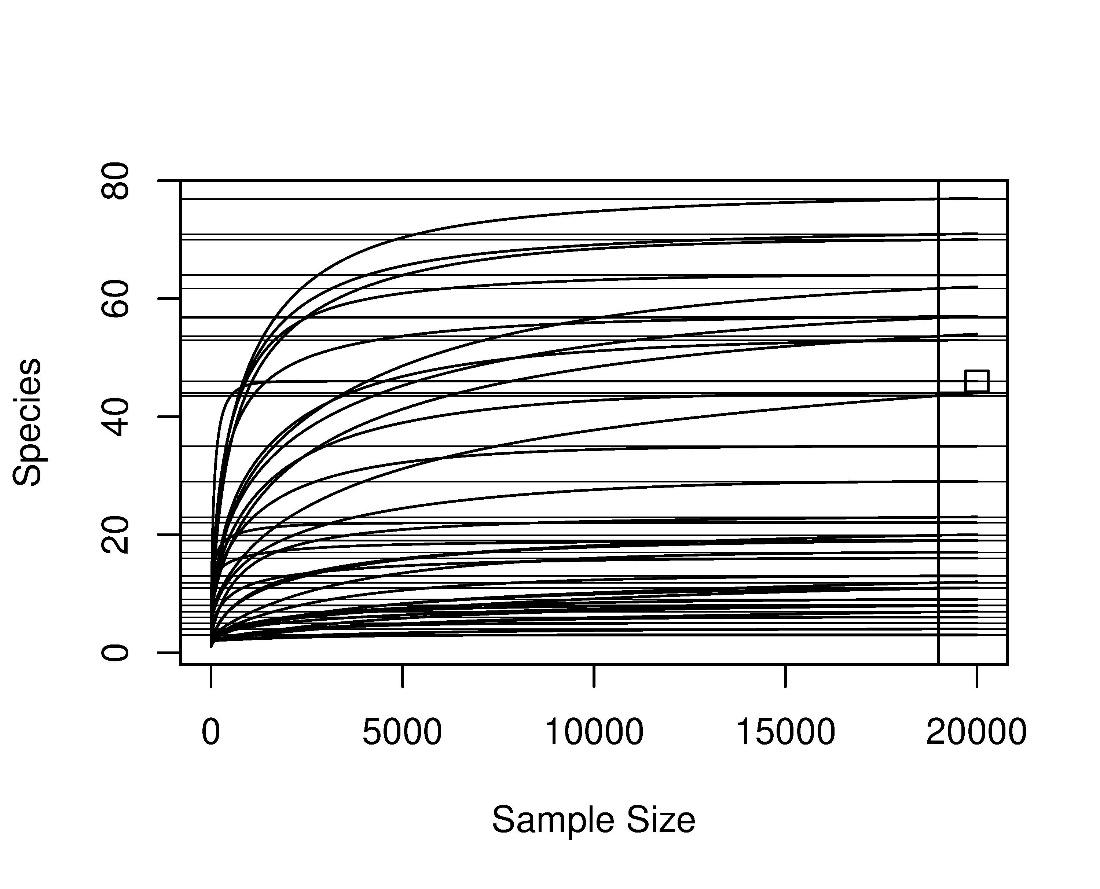


**Fig S5A.**


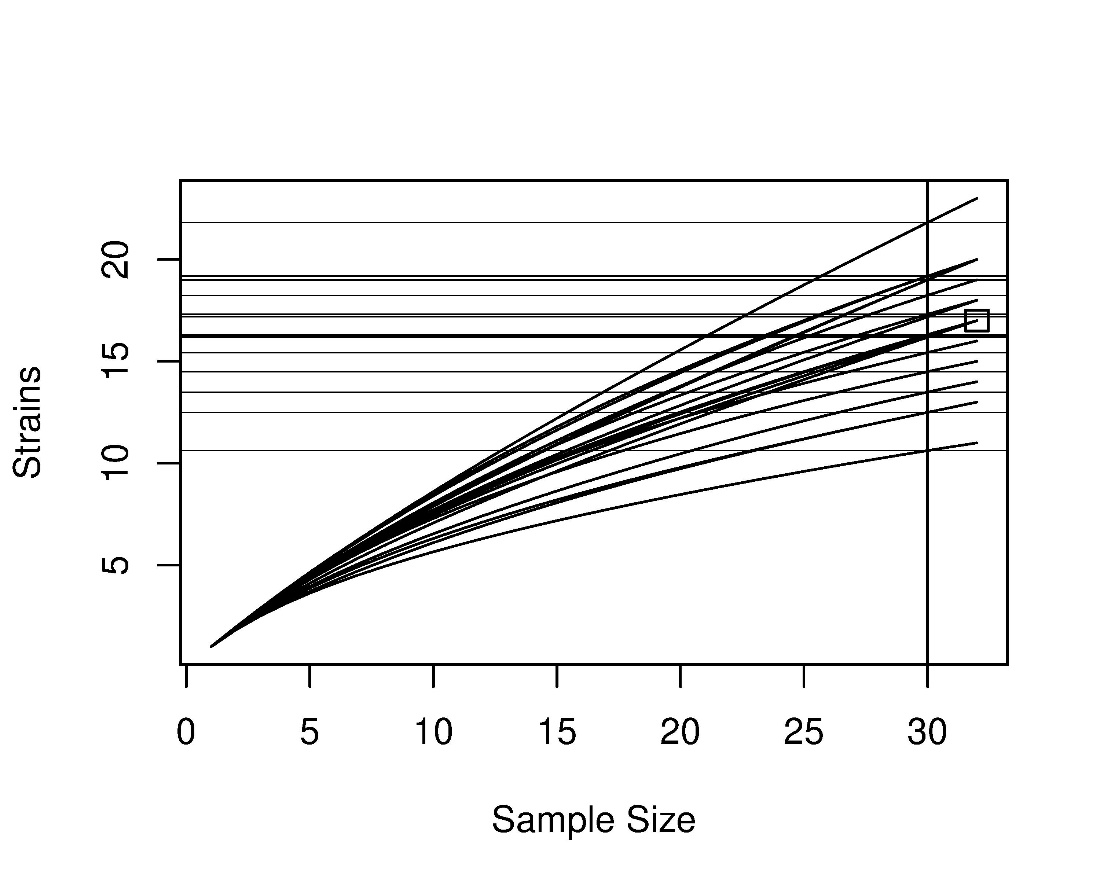


**Fig S5B.**

Supplement: S5 Fig — Rarefaction curves featuring (A) species richness in the fungal community, and (B) strain richness in the S. uvarum community, at different sampling depths. (DOCX) [file pone.0225615.s005.docx]
