# Supplementary material for: An indigenous Saccharomyces uvarum population with high genetic diversity dominates uninoculated Chardonnay fermentations at a Canadian winery
Source: PLoS One. 2021 Feb 4;16(2):e0225615. doi: 10.1371/journal.pone.0225615 (PMC7861373; doi:10.1371/journal.pone.0225615)
Supplement: S1 Table — Any significant differences (p ≤ 0.05) are in bold. (DOCX) [file pone.0225615.s006.docx]

**S1 Table.**

| **Stage** | **Chemical parameter** | ***F* (1,4) =** | ***p* =** |
| --- | --- | --- | --- |
| Cold settling | pH | 0.50 | 0.52 |
|  | Residual sugar (°Brix) | 1.00 | 0.37 |
|  | Yeast assimilable nitrogen (mg/L) | 0.17 | 0.70 |
|  | Titratable acidity (g/L) | 1.00 | 0.37 |
|  | Malic acid (g/L) | 1.00 | 0.37 |
| Late | pH | 0.10 | 0.77 |
|  | Titratable acidity (g/L) | 1.14 | 0.35 |
|  | Malic acid (g/L) | 0.50 | 0.52 |
|  | Volatile acidity (g/L) | 0.13 | 0.35 |
|  | Ethanol (% v/v) | 0.06 | 0.81 |
|  | Glucose (g/L) | 0.81 | 0.42 |
|  | Fructose (g/L) | 0.19 | 0.69 |
