# Supplementary material for: An indigenous Saccharomyces uvarum population with high genetic diversity dominates uninoculated Chardonnay fermentations at a Canadian winery
Source: PLoS One. 2021 Feb 4;16(2):e0225615. doi: 10.1371/journal.pone.0225615 (PMC7861373; doi:10.1371/journal.pone.0225615)
Supplement: S2 Table — Samples were taken from grapes in the vineyard (G), and at four stages of fermentation in the winey: cold settling (C), early (E), mid (M), and late (L). Vineyard 2 grape sample values are the means ± SEM of five replicates, and Vineyard 8 grape sample values are the means ± SEM of 6 replicates. All winery fermentation stages have three reported replicates, with the exception of the cold settling stage from the Vineyard 8 fermentations, which contained two. Sequences were identified to the species level unless otherwise indicated. Fungal species that represented less than 10% of the relative abundance in at least two samples were grouped into the Minor Fungi category. One exception was Saccharomyces cerevisiae, which never reached 10% relative abundance in any sample but is included in this table because of its importance during alcoholic fermentation. The last two columns indicate positive (Pos) and negative (Neg) controls. For the raw data containing all the fungi identified in this study (including minor fungi), please visit https://osf.io/j7rx8/. (DOCX) [file pone.0225615.s007.docx]

**S2 Table.**

|  | Vineyard 2 | | | | | Vineyard 8 | | | | | Controls | |
| --- | --- | --- | --- | --- | --- | --- | --- | --- | --- | --- | --- | --- |
| Fungi | G | C | E | M | L | G | C | E | M | L | Pos | Neg |
| *Saccharomyces uvarum* | 2.61  ±  2.6 | 0.35  ±  0.04 | 96.73  ±  0.3 | 96.88  ±  0.5 | 96.84  ±  0.5 | 0.027  ±  0.02 | 16.26  ±  2.8 | 87.47  ±  5.5 | 81.47  ±  2.9 | 79.71  ±  9.5 | 0 | 3.54 |
| *Erysiphe necator* | 1.09  ±  0.6 | 0.013  ±  0.01 | 0.0017  ±  0.001 | 0  ±  0 | 0.0033  ±  0.003 | 45.7  ±  7.0 | 0.020  ±  0.02 | 0.0050  ±  0.003 | 0  ±  0 | 0.0067  ±  0.006 | 0 | 10.73 |
| *Mycosphaerella tassiana* | 19.28  ±  4.1 | 0.12  ±  0.01 | 0.013  ±  0.007 | 0  ±  0 | 0  ±  0 | 17.4  ±  4.8 | 0.14  ±  0.1 | 0.0067  ±  0.003 | 0.40  ±  0.4 | 0  ±  0 | 0 | 0 |
| *Aspergillus niger* | 0  ±  0 | 56.65  ±  3.4 | 0.37  ±  0.06 | 0.083  ±  0.008 | 0.010  ±  0.01 | 0.0092  ±  0.007 | 0.070  ±  0.07 | 0  ±  0 | 0  ±  0 | 0  ±  0 | 0 | 0 |
| *Alternaria* sp. | 24.10  ±  4.9 | 0.12  ±  0.06 | 0.0050  ±  0.003 | 0  ±  0 | 0  ±  0 | 7.28  ±  1.4 | 0  ±  0 | 0  ±  0 | 0  ±  0 | 0  ±  0 | 0 | 0 |
| *Hanseniaspora osmophila* | 0  ±  0 | 0.10  ±  0.06 | 0.013  ±  0.01 | 0.030  ±  0.003 | 0  ±  0 | 0  ±  0 | 10.81  ±  4.2 | 11.59  ±  5.4 | 15.1  ±  3.4 | 16.98  ±  8.7 | 0 | 3.72 |
| *Aureobasidium pullulans* | 5.68  ±  1.4 | 12.65  ±  0.5 | 0.040  ±  0.006 | 0  ±  0 | 0.012  ±  0.01 | 8.05  ±  1.7 | 0.85  ±  0.1 | 0  ±  0 | 0.097  ±  0.06 | 0.032  ±  0.004 | 0 | 7.84 |
| *Epicoccum nigrum* | 14.52  ±  3.0 | 0.20  ±  0.2 | 0.0017  ±  0.001 | 0  ±  0 | 0  ±  0 | 5.42  ±  1.4 | 0  ±  0 | 0  ±  0 | 0.0017  ±  0.001 | 0  ±  0 | 0 | 0 |
| *Cladosporium* sp. | 0.89  ±  0.1 | 0.075  ±  0.04 | 0.0033  ±  0.003 | 0  ±  0 | 0.0050  ±  0.005 | 8.35  ±  4.9 | 1.90  ±  0.3 | 0  ±  0 | 0.0033  ±  0.003 | 0.0083  ±  0.004 | 0 | 0 |
| *Penicillium* sp. | 0.20  ±  0.2 | 8.33  ±  1.3 | 0.033  ±  0.004 | 0  ±  0 | 0  ±  0 | 0.78  ±  0.5 | 11.74  ±  6.4 | 0.0033  ±  0.003 | 0.18  ±  0.1 | 0  ±  0 | 0 | 0 |
| *Candida* sp. | 0  ±  0 | 0.47  ±  0.2 | 0.017  ±  0.009 | 0  ±  0 | 0  ±  0 | 0  ±  0 | 23.45  ±  1.2 | 0.0067  ±  0.004 | 0.047  ±  0.01 | 0.083  ±  0.06 | 0 | 0 |
| *Saccharomyces cerevisiae* | 1.61  ±  1.3 | 0.060  ±  0.03 | 2.68  ±  1.9 | 3.04  ±  0.5 | 3.04  ±  0.5 | 0.053  ±  0.03 | 6.11  ±  1.2 | 0.73  ±  0.086 | 0.84  ±  0.5 | 1.11  ±  0.4 | 99.99 | 0.17 |
| Minor Fungi | 30.03  ±  7.9 | 20.86  ±  1.8 | 0.11  ±  0.02 | 0.045  ±  0.03 | 0.097  ±  0.04 | 6.92  ±  1.1 | 28.67  ±  4.5 | 0.19  ±  0.02 | 1.86  ±  1.0 | 2.08  ±  1.4 | 0.010 | 74.02 |
