# Supplementary material for: An indigenous Saccharomyces uvarum population with high genetic diversity dominates uninoculated Chardonnay fermentations at a Canadian winery
Source: PLoS One. 2021 Feb 4;16(2):e0225615. doi: 10.1371/journal.pone.0225615 (PMC7861373; doi:10.1371/journal.pone.0225615)
Supplement: S3 Table — Allele sizes for allele 1 (A1) and allele 2 (A2) are shown for each of the 11 microsatellite loci analyzed. Strains with the prefix “2017” were isolated exclusively during the 2017 vintage. Strains with the prefix “2015” were previously isolated and characterized during the 2015 vintage at the same winery, and were also isolated during the 2017 vintage. Strains without a vintage prefix are those that belong to global yeast databases (see S5 Table). (DOCX) [file pone.0225615.s008.docx]

**S3 Table**.

|  | NB9 | | L1 | | L9 | | NB1 | | L8 | | L3 | | L7 | | L2 | | L4 | | NB4 | | NB8 | |
| --- | --- | --- | --- | --- | --- | --- | --- | --- | --- | --- | --- | --- | --- | --- | --- | --- | --- | --- | --- | --- | --- | --- |
| Strain Identity | A1 | A2 | A1 | A2 | A1 | A2 | A1 | A2 | A1 | A2 | A1 | A2 | A1 | A2 | A1 | A2 | A1 | A2 | A1 | A2 | A1 | A2 |
| 2017_004 | 88 | 88 | 167 | 167 | 271 | 271 | 206 | 206 | 220 | 220 | 228 | 228 | 259 | 259 | 284 | 284 | 318 | 318 | 350 | 350 | 439 | 439 |
| 2017_041 | 114 | 114 | 163 | 163 | 217 | 217 | 209 | 209 | 208 | 247 | 228 | 228 | 269 | 273 | 296 | 296 | 306 | 318 | 335 | 335 | 412 | 448 |
| 2017_081 | 114 | 114 | 167 | 167 | 280 | 280 | 212 | 212 | 247 | 247 | 224 | 224 | 267 | 267 | 299 | 299 | 300 | 300 | 335 | 335 | 412 | 412 |
| 2017_086 | 114 | 114 | 167 | 167 | 274 | 274 | 209 | 209 | 208 | 208 | 214 | 214 | 259 | 259 | 287 | 287 | 300 | 300 | 335 | 335 | 412 | 412 |
| 2017_095 | 114 | 114 | 167 | 167 | 172 | 217 | 209 | 212 | 208 | 208 | 224 | 228 | 259 | 269 | 287 | 287 | 306 | 318 | 335 | 344 | 439 | 439 |
| 2017_096 | 114 | 114 | 167 | 167 | 172 | 217 | 209 | 209 | 208 | 208 | 214 | 214 | 259 | 269 | 287 | 287 | 300 | 306 | 335 | 344 | 412 | 439 |
| 2017_097 | 114 | 114 | 167 | 167 | 172 | 217 | 209 | 209 | 208 | 208 | 224 | 224 | 259 | 269 | 287 | 287 | 306 | 306 | 335 | 344 | 439 | 439 |
| 2017_098 | 114 | 114 | 167 | 167 | 172 | 217 | 209 | 209 | 208 | 208 | 224 | 228 | 259 | 269 | 287 | 287 | 306 | 306 | 335 | 344 | 439 | 439 |
| 2017_101 | 114 | 114 | 167 | 167 | 217 | 220 | 206 | 209 | 208 | 220 | 218 | 224 | 259 | 269 | 287 | 296 | 300 | 306 | 335 | 350 | 412 | 439 |
| 2017_105 | 114 | 114 | 167 | 167 | 217 | 217 | 206 | 209 | 208 | 220 | 218 | 224 | 259 | 269 | 287 | 287 | 300 | 306 | 335 | 350 | 412 | 439 |
| 2017_107 | 114 | 114 | 167 | 167 | 271 | 271 | 209 | 212 | 208 | 208 | 214 | 214 | 259 | 259 | 287 | 287 | 300 | 300 | 335 | 335 | 412 | 412 |
| 2017_110 | 114 | 114 | 167 | 167 | 271 | 271 | 209 | 209 | 208 | 208 | 214 | 214 | 259 | 259 | 287 | 287 | 300 | 318 | 335 | 335 | 412 | 412 |
| 2017_111 | 114 | 114 | 167 | 167 | 271 | 271 | 209 | 209 | 208 | 208 | 214 | 214 | 259 | 259 | 287 | 287 | 300 | 300 | 335 | 335 | 412 | 412 |
| 2017_147 | 114 | 114 | 165 | 165 | 181 | 217 | 209 | 209 | 211 | 247 | 224 | 228 | 259 | 273 | 287 | 299 | 318 | 318 | 335 | 350 | 412 | 412 |
| 2017_148 | 114 | 114 | 165 | 165 | 217 | 220 | 209 | 209 | 247 | 247 | 228 | 228 | 273 | 273 | 299 | 299 | 318 | 318 | 335 | 335 | 412 | 412 |
| 2017_150 | 114 | 114 | 165 | 165 | 217 | 217 | 209 | 209 | 247 | 247 | 228 | 228 | 273 | 273 | 299 | 299 | 318 | 318 | 335 | 335 | 412 | 439 |
| 2017_151 | 114 | 114 | 165 | 165 | 217 | 217 | 209 | 209 | 247 | 247 | 228 | 228 | 273 | 273 | 299 | 299 | 318 | 318 | 335 | 335 | 412 | 412 |
| 2017_155 | 114 | 114 | 165 | 165 | 217 | 217 | 206 | 206 | 247 | 247 | 228 | 228 | 273 | 273 | 299 | 299 | 318 | 318 | 335 | 335 | 439 | 439 |
| 2017_168 | 114 | 122 | 163 | 167 | 181 | 181 | 212 | 212 | 208 | 208 | 218 | 224 | 269 | 269 | 287 | 296 | 318 | 318 | 350 | 350 | 448 | 448 |
| 2017_172 | 114 | 122 | 163 | 167 | 172 | 217 | 209 | 209 | 208 | 208 | 214 | 214 | 259 | 269 | 287 | 287 | 306 | 318 | 335 | 344 | 412 | 439 |
| 2017_173 | 114 | 122 | 163 | 167 | 172 | 217 | 209 | 209 | 208 | 208 | 224 | 228 | 259 | 269 | 287 | 287 | 306 | 306 | 335 | 344 | 439 | 439 |
| 2017_179 | 114 | 122 | 167 | 167 | 172 | 172 | 209 | 212 | 208 | 208 | 224 | 228 | 259 | 269 | 287 | 287 | 306 | 306 | 335 | 344 | 439 | 439 |
| 2017_180 | 114 | 122 | 167 | 167 | 172 | 172 | 209 | 209 | 208 | 208 | 224 | 228 | 259 | 269 | 287 | 287 | 306 | 306 | 335 | 344 | 439 | 439 |
| 2017_181 | 114 | 122 | 167 | 167 | 172 | 217 | 209 | 212 | 208 | 208 | 224 | 224 | 259 | 269 | 287 | 287 | 306 | 306 | 335 | 344 | 439 | 439 |
| 2017_182 | 114 | 122 | 167 | 167 | 172 | 217 | 209 | 209 | 208 | 208 | 224 | 224 | 259 | 269 | 287 | 287 | 306 | 306 | 335 | 344 | 439 | 439 |
| 2017_183 | 114 | 122 | 167 | 167 | 172 | 217 | 209 | 209 | 208 | 208 | 224 | 224 | 259 | 259 | 287 | 287 | 306 | 306 | 335 | 344 | 439 | 439 |
| 2017_184 | 114 | 122 | 167 | 167 | 172 | 217 | 209 | 209 | 208 | 208 | 224 | 228 | 259 | 269 | 287 | 287 | 306 | 306 | 335 | 344 | 439 | 439 |
| 2017_188 | 114 | 122 | 163 | 165 | 181 | 217 | 206 | 209 | 208 | 220 | 214 | 214 | 259 | 269 | 284 | 299 | 300 | 306 | 335 | 350 | 412 | 439 |
| 2017_193 | 114 | 122 | 165 | 167 | 172 | 217 | 209 | 209 | 208 | 208 | 218 | 224 | 259 | 269 | 287 | 287 | 306 | 318 | 335 | 344 | 412 | 439 |
| 2017_197 | 114 | 122 | 165 | 165 | 172 | 172 | 206 | 206 | 208 | 208 | 224 | 228 | 259 | 269 | 287 | 287 | 306 | 306 | 335 | 344 | 439 | 439 |
| 2017_224 | 122 | 122 | 167 | 167 | 316 | 316 | 206 | 206 | 208 | 208 | 218 | 218 | 269 | 269 | 287 | 287 | 306 | 306 | 350 | 350 | 439 | 439 |
| 2017_246 | 122 | 122 | 167 | 167 | 283 | 283 | 209 | 209 | 208 | 208 | 218 | 218 | 269 | 269 | 287 | 287 | 318 | 318 | 344 | 344 | 448 | 448 |
| 2017_300 | 122 | 122 | 165 | 165 | 295 | 295 | 206 | 206 | 214 | 214 | 218 | 218 | 269 | 269 | 287 | 287 | 300 | 300 | 350 | 350 | 448 | 448 |
| 2017_326 | 122 | 122 | 165 | 165 | 181 | 181 | 209 | 209 | 208 | 208 | 224 | 224 | 259 | 259 | 287 | 287 | 318 | 318 | 344 | 344 | 439 | 439 |
| 2017_337 | 122 | 122 | 165 | 165 | 286 | 286 | 212 | 212 | 208 | 208 | 218 | 228 | 269 | 269 | 287 | 287 | 300 | 318 | 335 | 350 | 412 | 448 |
| 2017_338 | 114 | 114 | 163 | 163 | 181 | 181 | 206 | 206 | 211 | 211 | 224 | 224 | 259 | 259 | 287 | 287 | 318 | 318 | 350 | 350 | 448 | 448 |
| 2015_017 | 114 | 122 | 163 | 167 | 274 | 280 | 206 | 206 | 214 | 220 | 228 | 228 | 259 | 259 | 293 | 305 | 318 | 318 | 350 | 350 | 439 | 439 |
| 2015_022 | 114 | 114 | 167 | 167 | 289 | 289 | 209 | 209 | 208 | 208 | 218 | 218 | 259 | 259 | 305 | 305 | 318 | 318 | 344 | 344 | 448 | 448 |
| 2015_030 | 114 | 114 | 167 | 167 | 292 | 292 | 209 | 209 | 208 | 208 | 218 | 218 | 269 | 269 | 305 | 305 | 318 | 318 | 335 | 335 | 448 | 448 |
| 2015_031 | 114 | 114 | 167 | 167 | 295 | 295 | 206 | 209 | 208 | 220 | 218 | 218 | 259 | 269 | 305 | 305 | 300 | 318 | 344 | 350 | 412 | 448 |
| 2015_034 | 114 | 114 | 167 | 167 | 271 | 271 | 206 | 206 | 214 | 214 | 228 | 228 | 269 | 269 | 293 | 293 | 318 | 318 | 350 | 350 | 448 | 448 |
| 2015_035 | 114 | 114 | 167 | 167 | 286 | 286 | 206 | 206 | 208 | 208 | 218 | 218 | 259 | 259 | 305 | 305 | 300 | 300 | 350 | 350 | 448 | 448 |
| 2015_042 | 114 | 114 | 167 | 167 | 187 | 187 | 209 | 209 | 208 | 208 | 228 | 228 | 269 | 269 | 293 | 293 | 306 | 306 | 335 | 335 | 448 | 448 |
| 2015_051 | 122 | 122 | 167 | 167 | 223 | 223 | 212 | 212 | 214 | 214 | 218 | 218 | 269 | 269 | 305 | 305 | 300 | 300 | 335 | 335 | 412 | 412 |
| 2015_055 | 114 | 114 | 167 | 167 | 187 | 187 | 212 | 212 | 208 | 208 | 218 | 218 | 259 | 259 | 293 | 293 | 318 | 318 | 335 | 335 | 448 | 448 |
| 2015_063 | 114 | 114 | 167 | 167 | 223 | 223 | 209 | 209 | 220 | 220 | 218 | 218 | 269 | 269 | 305 | 305 | 318 | 318 | 344 | 344 | 448 | 448 |
| 2015_078 | 114 | 114 | 163 | 167 | 187 | 277 | 206 | 209 | 220 | 220 | 214 | 214 | 259 | 259 | 293 | 305 | 300 | 318 | 335 | 350 | 439 | 448 |
| 2015_086 | 114 | 114 | 163 | 163 | 187 | 187 | 206 | 206 | 211 | 220 | 224 | 224 | 259 | 259 | 293 | 293 | 306 | 318 | 335 | 350 | 439 | 448 |
| 2015_094 | 114 | 114 | 163 | 163 | 283 | 283 | 206 | 206 | 229 | 229 | 218 | 218 | 269 | 269 | 290 | 290 | 306 | 306 | 350 | 350 | 448 | 448 |
| 2015_117 | 114 | 114 | 163 | 163 | 187 | 187 | 212 | 212 | 220 | 220 | 218 | 218 | 269 | 269 | 290 | 290 | 318 | 318 | 335 | 335 | 448 | 448 |
| 2015_128 | 114 | 114 | 163 | 163 | 280 | 280 | 212 | 212 | 220 | 220 | 218 | 218 | 259 | 259 | 293 | 293 | 318 | 318 | 335 | 335 | 439 | 439 |
| 2015_133 | 114 | 114 | 163 | 163 | 277 | 289 | 206 | 209 | 208 | 208 | 218 | 228 | 259 | 259 | 305 | 305 | 300 | 318 | 344 | 350 | 439 | 448 |
| 2015_136 | 114 | 114 | 163 | 163 | 277 | 277 | 212 | 212 | 220 | 220 | 228 | 228 | 259 | 259 | 293 | 293 | 300 | 300 | 335 | 335 | 439 | 439 |
| 2015_151 | 114 | 122 | 163 | 167 | 277 | 277 | 206 | 206 | 208 | 220 | 228 | 228 | 259 | 259 | 293 | 305 | 300 | 318 | 350 | 350 | 439 | 448 |
| 2015_163 | 114 | 114 | 163 | 163 | 223 | 281 | 206 | 209 | 208 | 208 | 218 | 228 | 259 | 269 | 293 | 302 | 306 | 306 | 335 | 350 | 439 | 448 |
| 2015_193 | 114 | 114 | 163 | 163 | 223 | 223 | 206 | 206 | 208 | 208 | 228 | 228 | 269 | 269 | 305 | 305 | 300 | 300 | 350 | 350 | 439 | 439 |
| 2015_214 | 114 | 114 | 165 | 165 | 226 | 226 | 209 | 209 | 208 | 208 | 224 | 224 | 269 | 269 | 302 | 302 | 306 | 306 | 335 | 335 | 412 | 412 |
| 2015_217 | 114 | 114 | 165 | 165 | 289 | 289 | 212 | 212 | 208 | 208 | 228 | 228 | 269 | 269 | 305 | 305 | 300 | 300 | 350 | 350 | 448 | 448 |
| 2015_221 | 114 | 114 | 163 | 163 | 277 | 277 | 206 | 206 | 220 | 220 | 228 | 228 | 259 | 259 | 293 | 293 | 300 | 300 | 350 | 350 | 439 | 439 |
| 2015_233 | 114 | 114 | 165 | 165 | 277 | 289 | 212 | 212 | 208 | 220 | 218 | 228 | 259 | 269 | 305 | 305 | 300 | 300 | 335 | 335 | 412 | 439 |
| 2015_245 | 114 | 114 | 165 | 165 | 223 | 223 | 206 | 209 | 208 | 208 | 224 | 228 | 267 | 269 | 302 | 305 | 300 | 306 | 335 | 350 | 412 | 448 |
| 2015_260 | 114 | 114 | 165 | 165 | 223 | 223 | 206 | 206 | 220 | 220 | 224 | 224 | 267 | 267 | 305 | 305 | 300 | 300 | 350 | 350 | 412 | 412 |
| 2015_271 | 122 | 122 | 167 | 167 | 223 | 271 | 206 | 206 | 214 | 214 | 228 | 228 | 269 | 269 | 293 | 293 | 300 | 300 | 350 | 350 | 439 | 439 |
| 2015_274 | 114 | 122 | 163 | 167 | 271 | 280 | 206 | 206 | 208 | 220 | 228 | 228 | 259 | 259 | 293 | 305 | 300 | 300 | 350 | 350 | 439 | 439 |
| 2015_283 | 122 | 122 | 167 | 167 | 292 | 292 | 212 | 212 | 208 | 208 | 218 | 228 | 269 | 269 | 293 | 293 | 300 | 300 | 335 | 350 | 412 | 448 |
| 2015_288 | 114 | 114 | 167 | 167 | 223 | 223 | 212 | 212 | 214 | 214 | 224 | 224 | 259 | 259 | 293 | 293 | 306 | 306 | 335 | 335 | 412 | 412 |
| 2015_290 | 114 | 122 | 163 | 167 | 241 | 274 | 206 | 212 | 208 | 220 | 218 | 228 | 259 | 269 | 293 | 293 | 300 | 318 | 335 | 350 | 439 | 448 |
| 2015_308 | 114 | 122 | 163 | 167 | 223 | 277 | 209 | 209 | 208 | 208 | 228 | 228 | 269 | 269 | 293 | 302 | 306 | 318 | 335 | 350 | 412 | 448 |
| 2015_322 | 114 | 122 | 163 | 165 | 271 | 277 | 206 | 206 | 208 | 220 | 228 | 228 | 259 | 269 | 293 | 305 | 300 | 339 | 350 | 350 | 439 | 439 |
| 2015_333 | 122 | 122 | 165 | 165 | 277 | 295 | 209 | 209 | 208 | 208 | 218 | 228 | 267 | 267 | 293 | 305 | 300 | 318 | 335 | 350 | 412 | 448 |
| 2015_345 | 122 | 122 | 165 | 165 | 277 | 277 | 206 | 206 | 214 | 214 | 228 | 228 | 269 | 269 | 293 | 293 | 318 | 318 | 350 | 350 | 439 | 439 |
| 2015_363 | 122 | 122 | 167 | 167 | 277 | 277 | 212 | 212 | 208 | 208 | 218 | 228 | 269 | 269 | 305 | 305 | 300 | 300 | 335 | 335 | 412 | 412 |
| 2015_378 | 122 | 122 | 165 | 165 | 271 | 289 | 206 | 206 | 208 | 214 | 228 | 228 | 259 | 259 | 293 | 293 | 300 | 300 | 350 | 350 | 439 | 448 |
| 2015_390 | 122 | 122 | 151 | 165 | 292 | 292 | 212 | 212 | 208 | 208 | 218 | 228 | 269 | 269 | 293 | 305 | 300 | 318 | 335 | 350 | 412 | 448 |
| 2015_427 | 122 | 122 | 167 | 167 | 187 | 187 | 206 | 206 | 220 | 220 | 218 | 218 | 259 | 259 | 305 | 305 | 300 | 300 | 350 | 350 | 439 | 439 |
| 2015_433 | 122 | 122 | 167 | 167 | 277 | 292 | 212 | 212 | 208 | 208 | 218 | 228 | 269 | 269 | 293 | 293 | 300 | 318 | 350 | 350 | 412 | 448 |
| 2015_444 | 122 | 122 | 165 | 165 | 277 | 277 | 212 | 212 | 208 | 208 | 218 | 228 | 269 | 269 | 293 | 305 | 300 | 300 | 335 | 350 | 412 | 412 |
| 2015_449 | 122 | 122 | 167 | 167 | 277 | 277 | 206 | 206 | 208 | 208 | 228 | 228 | 259 | 259 | 293 | 293 | 300 | 300 | 350 | 350 | 448 | 448 |
| 2015_451 | 122 | 122 | 167 | 167 | 187 | 277 | 212 | 212 | 208 | 208 | 214 | 218 | 269 | 269 | 293 | 293 | 318 | 318 | 350 | 350 | 412 | 448 |
| 2015_454 | 122 | 122 | 167 | 167 | 277 | 277 | 212 | 212 | 208 | 208 | 218 | 218 | 269 | 269 | 293 | 293 | 318 | 318 | 335 | 335 | 412 | 412 |
| 2015_465 | 122 | 122 | 167 | 167 | 277 | 277 | 212 | 212 | 208 | 208 | 228 | 228 | 269 | 269 | 305 | 305 | 300 | 300 | 350 | 350 | 412 | 412 |
| 2015_496 | 122 | 122 | 167 | 167 | 226 | 226 | 206 | 206 | 208 | 208 | 228 | 228 | 269 | 269 | 293 | 293 | 318 | 318 | 350 | 350 | 412 | 412 |
| 2015_506 | 122 | 122 | 163 | 163 | 280 | 280 | 209 | 209 | 208 | 208 | 224 | 224 | 259 | 259 | 293 | 293 | 318 | 318 | 335 | 335 | 448 | 448 |
| 2015_518 | 122 | 122 | 165 | 165 | 277 | 277 | 206 | 206 | 214 | 214 | 228 | 228 | 259 | 259 | 293 | 293 | 318 | 318 | 350 | 350 | 439 | 439 |
| 2015_524 | 122 | 122 | 163 | 163 | 223 | 223 | 212 | 212 | 220 | 220 | 224 | 224 | 267 | 267 | 293 | 293 | 300 | 300 | 335 | 335 | 439 | 439 |
| 2015_541 | 122 | 122 | 165 | 165 | 223 | 271 | 206 | 206 | 208 | 208 | 228 | 228 | 259 | 269 | 293 | 305 | 300 | 339 | 350 | 350 | 439 | 448 |
| 2015_567 | 122 | 122 | 165 | 165 | 292 | 292 | 212 | 212 | 208 | 208 | 218 | 218 | 269 | 269 | 293 | 293 | 300 | 318 | 335 | 335 | 412 | 448 |
| 2015_595 | 122 | 122 | 165 | 165 | 292 | 292 | 212 | 212 | 208 | 208 | 218 | 218 | 269 | 269 | 293 | 293 | 318 | 318 | 335 | 335 | 448 | 448 |
| 2015_622 | 122 | 122 | 165 | 165 | 271 | 292 | 206 | 212 | 208 | 214 | 218 | 218 | 269 | 269 | 293 | 293 | 300 | 318 | 335 | 350 | 439 | 448 |
| 2015_659 | 122 | 122 | 167 | 167 | 271 | 271 | 206 | 206 | 214 | 214 | 228 | 228 | 269 | 269 | 293 | 293 | 318 | 318 | 350 | 350 | 448 | 448 |
| 2015_677 | 122 | 122 | 165 | 165 | 292 | 292 | 212 | 212 | 208 | 208 | 228 | 228 | 269 | 269 | 293 | 293 | 300 | 318 | 350 | 350 | 412 | 412 |
| 2015_694 | 122 | 122 | 165 | 165 | 292 | 292 | 212 | 212 | 208 | 208 | 228 | 228 | 269 | 269 | 293 | 293 | 300 | 300 | 335 | 335 | 412 | 412 |
| 2015_698 | 98 | 122 | 167 | 167 | 277 | 292 | 212 | 212 | 208 | 208 | 218 | 228 | 269 | 269 | 293 | 305 | 318 | 318 | 335 | 350 | 412 | 448 |
| 2015_706 | 122 | 122 | 165 | 165 | 274 | 274 | 212 | 212 | 208 | 208 | 228 | 228 | 269 | 269 | 302 | 302 | 300 | 300 | 350 | 350 | 412 | 412 |
| 2015_713 | 122 | 122 | 167 | 167 | 277 | 277 | 212 | 212 | 208 | 208 | 218 | 218 | 269 | 269 | 305 | 305 | 318 | 318 | 350 | 350 | 412 | 412 |
| 2015_743 | 122 | 122 | 165 | 165 | 277 | 277 | 206 | 206 | 220 | 220 | 224 | 224 | 259 | 259 | 305 | 305 | 318 | 318 | 350 | 350 | 439 | 439 |
| 2015_768 | 122 | 122 | 163 | 163 | 223 | 223 | 209 | 209 | 208 | 208 | 224 | 224 | 269 | 269 | 290 | 290 | 306 | 306 | 335 | 335 | 412 | 412 |
| 2015_777 | 122 | 122 | 165 | 165 | 223 | 223 | 206 | 206 | 208 | 208 | 228 | 228 | 269 | 269 | 305 | 305 | 339 | 339 | 350 | 350 | 439 | 439 |
| 2015_strain_1 | 114 | 114 | 163 | 163 | 277 | 277 | 206 | 206 | 220 | 220 | 228 | 228 | 259 | 259 | 305 | 305 | 300 | 300 | 350 | 350 | 439 | 439 |
| 2015_strain_2 | 122 | 122 | 165 | 165 | 277 | 277 | 212 | 212 | 208 | 208 | 218 | 228 | 269 | 269 | 293 | 305 | 300 | 318 | 335 | 350 | 412 | 448 |
| 2015_strain_3 | 114 | 114 | 163 | 163 | 223 | 223 | 209 | 209 | 208 | 208 | 228 | 228 | 269 | 269 | 302 | 302 | 306 | 306 | 335 | 335 | 448 | 448 |
| 2015_strain_4 | 122 | 122 | 165 | 165 | 277 | 292 | 212 | 212 | 208 | 208 | 218 | 228 | 269 | 269 | 293 | 293 | 300 | 318 | 350 | 350 | 412 | 448 |
| PYCC6860 | 114 | 114 | 165 | 165 | 217 | 217 | 206 | 206 | 208 | 208 | 224 | 224 | 269 | 269 | 284 | 284 | 300 | 300 | 350 | 350 | 412 | 412 |
| BMV58 | 122 | 122 | 165 | 165 | 274 | 274 | 206 | 206 | 208 | 208 | 228 | 228 | 259 | 259 | 284 | 284 | 300 | 300 | 350 | 350 | 448 | 448 |
| CBS7001 | 122 | 122 | 165 | 165 | 283 | 283 | 206 | 206 | 208 | 208 | 228 | 228 | 269 | 269 | 287 | 287 | 318 | 318 | 350 | 350 | 439 | 439 |
| CBS8711 | 114 | 114 | 167 | 167 | 220 | 217 | 212 | 212 | 220 | 220 | 218 | 218 | 271 | 271 | 287 | 287 | 318 | 318 | 335 | 335 | 448 | 448 |
