# Supplementary material for: An indigenous Saccharomyces uvarum population with high genetic diversity dominates uninoculated Chardonnay fermentations at a Canadian winery
Source: PLoS One. 2021 Feb 4;16(2):e0225615. doi: 10.1371/journal.pone.0225615 (PMC7861373; doi:10.1371/journal.pone.0225615)
Supplement: S4 Table — Samples were taken at three stages of fermentation: early (E), mid (M), and late (L). Values are the means ± SEM (n = 3). Strains that represented less than 10% of the relative abundance in at least two samples were grouped into the Minor Strains category. For the raw data, containing all the S. uvarum strains identified in this study (including minor strains), please visit https://osf.io/j7rx8/. (DOCX) [file pone.0225615.s009.docx]

**S4 Table.**

|  | Vineyard 2 | | | Vineyard 8 | | |
| --- | --- | --- | --- | --- | --- | --- |
| Strain | E | M | L | E | M | L |
| 2015 Strain 3 | 21.9 ± 7.2 | 18.8 ± 6.5 | 19.8 ± 1.0 | 13.5 ± 2.1 | 14.6 ± 2.8 | 27.1 ± 6.8 |
| 2015 Strain 2 | 12.5 ± 1.8 | 17.7 ± 2.8 | 17.7 ± 9.9 | 11.5 ± 1.0 | 14.6 ± 2.8 | 9.38 ± 3.6 |
| 2017 Strain 151 | 13.5 ± 2.8 | 7.29 ± 3.8 | 16.7 ± 7.5 | 0 ± 0 | 1.04 ± 1.04 | 0 ± 0 |
| 2017 Strain 182 | 0 ± 0 | 0 ± 0 | 0 ± 0 | 2.08 ± 1.0 | 12.5 ± 7.2 | 3.13 ± 1.8 |
| Minor Strains | 52.1 ± 5.5 | 56.3 ± 4.8 | 45.8 ± 9.1 | 72.9 ± 2.8 | 57.3 ± 4.2 | 60.4 ± 7.5 |
