# Supplementary material for: An indigenous Saccharomyces uvarum population with high genetic diversity dominates uninoculated Chardonnay fermentations at a Canadian winery
Source: PLoS One. 2021 Feb 4;16(2):e0225615. doi: 10.1371/journal.pone.0225615 (PMC7861373; doi:10.1371/journal.pone.0225615)
Supplement: S5 Table — Allele sizes for allele 1 (A1) and allele 2 (A2) are shown for each of the 11 microsatellite loci. (DOCX) [file pone.0225615.s010.docx]

**S5 Table**.

|  |  | NB9 | | L1 | | L9 | | NB1 | | L8 | | L3 | | L7 | | L2 | | L4 | | NB4 | | NB8 | |
| --- | --- | --- | --- | --- | --- | --- | --- | --- | --- | --- | --- | --- | --- | --- | --- | --- | --- | --- | --- | --- | --- | --- | --- |
| Strain ID | Origin | A1 | A2 | A1 | A2 | A1 | A2 | A1 | A2 | A1 | A2 | A1 | A2 | A1 | A2 | A1 | A2 | A1 | A2 | A1 | A2 | A1 | A2 |
| CBS395 | Netherlands | 114 | 114 | 167 | 167 | 217 | 217 | 212 | 212 | 220 | 220 | 228 | 228 | 259 | 259 | 284 | 284 | 318 | 318 | 335 | 335 | 439 | 439 |
| PYCC6860 | Hornby Island (Canada) | 114 | 114 | 165 | 165 | 217 | 217 | 206 | 206 | 208 | 208 | 224 | 224 | 269 | 269 | 284 | 284 | 300 | 300 | 350 | 350 | 412 | 412 |
| PYCC6861 | Hornby Island (Canada) | 114 | 114 | 165 | 165 | 220 | 220 | 209 | 209 | 220 | 220 | 224 | 224 | 269 | 269 | 284 | 284 | 306 | 306 | 335 | 335 | 412 | 412 |
| CBS7001 | Spain | 122 | 122 | 165 | 165 | 283 | 283 | 206 | 206 | 208 | 208 | 228 | 228 | 269 | 269 | 287 | 287 | 318 | 318 | 350 | 350 | 439 | 439 |
| BMV58 | Spain | 122 | 122 | 165 | 165 | 274 | 274 | 206 | 206 | 208 | 208 | 228 | 228 | 259 | 259 | 284 | 284 | 300 | 300 | 350 | 350 | 448 | 448 |
| CBS8690 | Moldova | 122 | 122 | 167 | 167 | 223 | 223 | 206 | 206 | 214 | 214 | 228 | 228 | 271 | 271 | 284 | 284 | 300 | 300 | 350 | 350 | 439 | 439 |
| CBS8696 | California | 122 | 122 | 167 | 167 | 283 | 283 | 209 | 209 | 214 | 214 | 228 | 228 | 261 | 261 | 287 | 287 | 318 | 318 | 335 | 335 | 412 | 412 |
| CBS8711 | France | 114 | 114 | 167 | 167 | 220 | 217 | 212 | 212 | 220 | 220 | 218 | 218 | 271 | 271 | 287 | 287 | 318 | 318 | 335 | 335 | 448 | 448 |
| PYCC6862 | Japan | 114 | 114 | 165 | 165 | 178 | 178 | 206 | 206 | 214 | 214 | 218 | 218 | 271 | 271 | 287 | 287 | 318 | 318 | 350 | 350 | 442 | 442 |
| PYCC6871 | Portugal | 122 | 122 | 165 | 165 | 232 | 232 | 206 | 206 | 208 | 208 | 228 | 228 | 271 | 271 | 287 | 287 | 318 | 318 | 350 | 350 | 439 | 439 |
| PYCC6901 | Oregon | 122 | 122 | 165 | 165 | 283 | 283 | 209 | 209 | 214 | 214 | 228 | 228 | 261 | 261 | 300 | 300 | 318 | 318 | 335 | 335 | 412 | 412 |
| PYCC6902 | Missouri | 122 | 122 | 167 | 167 | 289 | 289 | 209 | 209 | 220 | 220 | 218 | 218 | 271 | 271 | 300 | 300 | 318 | 318 | 350 | 350 | 439 | 439 |
