# Supplementary material for: An indigenous Saccharomyces uvarum population with high genetic diversity dominates uninoculated Chardonnay fermentations at a Canadian winery
Source: PLoS One. 2021 Feb 4;16(2):e0225615. doi: 10.1371/journal.pone.0225615 (PMC7861373; doi:10.1371/journal.pone.0225615)
Supplement: Attachment — Submitted filename: Response to Reviewers.docx [file pone.0225615.s011.docx]

**Manuscript:** **An indigenous Saccharomyces uvarum population with high genetic diversity dominates uninoculated Chardonnay fermentations at a Canadian winery** **[PONE-D-19-30860]**

Reviewer and Editor comments:

**Please note that references to line numbers in the manuscript refer to the cleaned and highlighted version, not the track-changes version.*

**Associate Editor**

Thank you for submitting your manuscript to PLOS ONE. After careful consideration, we feel that it has merit but does not fully meet PLOS ONE’s publication criteria as it currently stands. Therefore, we invite you to submit a revised version of the manuscript that addresses the points raised during the review process, in particular the following major points raised by referees: 1- *S. cerevisiae* x *S. uvarum* hybrid detection, 2- the use of another software than adegenet for the detection of admixture, 3- statistical issues raised by second referee, such as Kruskal-Wallis text vs ANOVA, and statistical evaluation of communities, 4- taking into account heterozygosity at microsatellite loci. All minor points raised by referees should be addressed as well.

1. *S. cerevisiae x S. uvarum hybrids:*
   a. *A discussion of the potential for S. cerevisiae x S. uvarum hybrids in this population has been added on Lines 892-916. In this manuscript, we only present strains for which all 11 microsatellite loci amplify with S. uvarum primers, whereas typically hybrid strains have a reduced success rate of microsatellite amplification with a primer set for one particular species. In a previous study conducted in 2015 at the same winery (Morgan et al. 2019, DOI: 10.1093/femsyr/foz049), yeast isolates were initially presumed to belong to S. cerevisiae, but no amplification was detected with S. cerevisiae microsatellite primers, which is what initially led us to test S. uvarum primers on these isolates. Therefore, we know conclusively that the 66 strains identified in both the current study and the 2015 study are not hybrids with S. cerevisiae. Future studies targeting the presence of hybrid strains will help us determine if they do indeed constitute an important component of the yeast community at this winery, but investigating hybrid strains was not an objective in this study.*
   b. *The Wallerstein agar was determined previously to show a definitive colour difference between these two species (Morgan et al. 2020, DOI: 10.5344/ajev.2020.19072; also see Figure S2, newly added to this manuscript). Therefore, we chose this method to differentiate between presumed S. cerevisiae and presumed S. uvarum isolates prior to identification via microsatellite analysis.*
   c. *We are currently conducting whole genome sequencing of five of the strains identified in this study. We will be mapping the sequencing reads against the S. uvarum reference strain CBS 7001 in the coming month, which will allow us to conclusively determine how much of the genomes of these strains maps to S. uvarum, and what introgressions there may be from other Saccharomyces species. However, the whole genome sequencing project is beyond the scope of this current study.*
2. *At the request of both reviewers, we have added an analysis of population structure of the strains of significance as compared to international strains, using InStruct software and visualized with DISTRUCT plots. Related discussions can be viewed on Lines 808-848, and the plot is presented in a new figure (Fig 5).*
3. *Statistical analysis of the fungal communities and S. uvarum populations have been added. Details of the statistical tests are described in the completely-revised Statistical Analysis section of the Materials and Methods (Lines 337-429), while a discussion of statistical results has been added throughout the Results and Discussion section (Lines 496-499; 525-527; 595-598; 663-664; 729-732; and Tables 3, 4, and 5). We have chosen not to perform non-parametric tests such as Kruskal-Wallis tests, and instead to keep the parametric ANOVA tests, because our data met the assumption of homogeneity of variance, and ANOVA are robust to deviations from normality. We have explained our reasoning on Lines 342-344.*
4. *We have expanded our discussion of the genetic diversity and population structure of this yeast population, including fixation indices and observed vs. expected heterozygosity, on Lines 780-791; 803-807; 808-848.*

**Reviewer #1**

In this manuscript McCarthy et al. present the characterization of a unique yeast population from a Canadian winery. Interestingly, this winery is inhabited by a *S. uvarum* population, which makes it one of the few example of yeast microflora not containing *Saccharomyces cerevisiae*. It presents a comprehensive analysis of the fungal population from the vineyard to the vats, and an analysis of *S. uvarum* population in the winery and the vineyard.

This original, well led and well written study is an interesting case study of alternative micro-flora under cold climate. This case was known for cool climate vineyards, but until now, this is the first study presenting this situation.

Despite the quality of the manuscript, I feel one main lack and few missing piece of information or analyses.

1 – The authors have selected theirs strains on Wallerstein nutrient media. This is not a robust species identification method, and authors have not explored for the presence of *S. cerevisiae* x *S. uvarum* interspecific hybrids that may participate to the ecosystem. Doing so they may have omitted one component of the fermentation microflore. This had been described in one of the first winery containing S. uvarum strains (Demuyter et al. 2014 J. Applied Microbiol , LeJeune et al. 2007 FEMS Yeast Research), and I personally have detected many others in cold cellars. As a consequence, it is necessary to check for the presence of hybrids among the “*S. uvarum*” isolates (that microsatellite considered as *S. uvarum*) or among the *S. cerevisiae*. An overview of the frequencies of the counts of *S. uvarum* and *S. cerevisiae* in the different samples is missing.

- *The Wallerstein agar was determined previously to show a definitive colour difference between these two species (Morgan et al. 2020, DOI: 10.5344/ajev.2020.19072; also see Figure S2, newly added to this manuscript). Therefore, we chose this method to differentiate between presumed S. cerevisiae and presumed S. uvarum isolates prior to identification via microsatellite analysis.*
- *We have added a discussion of the potential for S. cerevisiae x S. uvarum hybrids in this population on Lines 892-916. In this manuscript, we only present strains for which all 11 microsatellite loci amplify with S. uvarum primers, whereas typically hybrid strains have a reduced success rate of microsatellite amplification with a primer set for one particular species. In a previous study conducted in 2015 at the same winery (Morgan et al. 2019, DOI: 10.1093/femsyr/foz049), yeast isolates were initially presumed to belong to S. cerevisiae, but no amplification was detected with S. cerevisiae microsatellite primers, which is what initially led us to test S. uvarum primers on these isolates. Therefore, we know that the 66 strains identified in both the current study and the 2015 study are not hybrids with S. cerevisiae. We did not test the isolates that only partially amplified using S. uvarum primers with S. cerevisiae primers; future studies targeting the presence of hybrid strains will help us determine if they do indeed constitute an important component of the yeast community at this winery. However, the targeting of hybrids was not an objective of this study.*
- *We have added a discussion of the LeJeune et al. 2007 paper that looked at hybrids (Lines 905-907), and we believe the Demuyter et al. 2014 paper mentioned was a typo, and refers to the Demuyter et al. 2004 paper, which is referenced throughout the manuscript.*
- *To acknowledge that there is the potential for hybrid or mosaic genomes, we have used the phrase ‘presumed S. cerevisiae’ and ‘presumed S. uvarum’ when referring to pre-strain typed yeast isolates. We have included two new figures (Fig 2 and Fig S2) that show the relative proportion of presumed S. cerevisiae and presumed S. uvarum strains in the fermentations, as estimated by visual differentiation on WLN media. The presumed S. cerevisiae isolates were in fact strain-typed (strain identities available on OSF:* *https://osf.io/j7rx8/). A discussion of these results has been added to Lines 648-652.*

2 – When analyzing their population structure, with the package adegenet, the authors mention that the topology of the tree was not matching the clustering obtained with the “find.cluster function of the adegenet package. This function is very efficient for the identification of groups, but less for the identification of admixed populations. Alternative populations structure software enable such inferences : i.e. InStruct, or Structure softwares take into account admixture, making the assumption of an inbred population (described as the case in manuscripts of other groups) or maximizing the likelihood of Hardy Weinberg equilibrium.

- *We have added an analysis of population structure of the strains of significance as compared to international strains, using InStruct software and visualized with DISTRUCT plots. Related discussions can be viewed on Lines 808-848, and the plot can be seen in Fig 5.*
- *We have also removed any statements regarding admixed or panmictic populations in reference to the tree (Fig 6), which has been updated with bootstrap values.*

3 – Some minors point

Line 127-147 : what is the total weight of the grape samples?

- *The total weight of the grape samples was 2-3 kg (Line 142).*

Line 364: the distance used for the estimation of dissimilarity is not indicated: the function “vegdist” of the vegan package uses several dissimilarity distances (i.e : Bray-Curtis, Jaccard…)

- *We apologize for this unintended omission. We have re-written the entire Statistical Analysis section, and have included this information. We used a Bray-Curtis dissimilarity matrix when performing PERMANOVA/PERMDISP tests (Lines 372, 376) and generating the PCoA ordination (Line 389).*

Line 424 : the unit in which volatile acidity is expressed is not indicated : is this g/L of acetic acid, or H_2_SO_4_? This threshold is very high and caused by the choice of the analytical method. Other methods (i.e. enzymatic kits, or distillation) provide much higher sensitivity.

- *Estimated as g/L acetic acid (added to Line 485).*
- *In this context, ‘detection threshold’ refers to the sensory (tasting) threshold, not an analytical detection threshold. This has been clarified in text (Line 488).*

Line 439-449: Table 4 : Why only Simpson’s Index is given. Other indices such as the popular Shannon index provide complementary information on the diversity isolated in the samples. A rarefaction method should be used in order to avoid variations of sample size (several tools/package enable such inferences i.e. EstimateS…)

- *We have added Shannon’s Index to Table 4 and Table 5. We initially chose Simpson’s index because it places more emphasis on dominant species/strains, which is what we were most interested in.*
- *We have created rarefaction curves for both the fungal species and S. uvarum strains (Lines 348-356; Fig S3). However, our data did not include variations of sample size during analysis, because all samples were rarefied prior to analysis.*

Line 523 : how far is vineyard 2 from the cellar (or from any other) in comparison to vineyard 1?

- *The two vineyards are directly south of the winery and separated from each other by approximately 30 km. This information has been added to Lines 119-121.*

Line 567-72 Table 5 : same comment as for table 4.

- *We have added Shannon’s Index to Table 4 and Table 5.*

Line 615-31: same as above : which dissimilarity distance has been used for PCoA

- *We used a Bray-Curtis dissimilarity matrix when performing PERMANOVA/PERMDISP tests (Lines 372, 376) and generating the PCoA ordination (Line 389).*

Line 654 : “genetically similar to the commercial strain Velluto BMV58” . Could the authors estimate a likelihood to obtain the same genotype by chance from the frequencies of each allele in this dataset ?

- *We have used GenAlEx to calculate probability of identity (P_I_) - the probability that two unrelated individuals would have identical multilocus genotypes. P_I_ was calculated to be 6.7 × 10^-8^. However, this probability does not take into account the Bruvo distance used in this study; individuals in this study could have slightly different MLGs and still be grouped into the same strain classification. This discussion has been added to Lines 780-784.*

Line 663-667 : more than the number of heterozygote loci per strains , or heterozygote strains, I think that it is more relevant to infer a selfing rate. It takes into account the allelic richness of the loci and make the comparison easier for different datasets.

- *We have used GenAlEx to calculate fixation indices (F_ST_ and F_IS_) which estimate the variance in allele frequency between subpopulations and inbreeding coefficients, respectively. F_ST_ was calculated to be 0.014 ± 0.003, indicating that the two subpopulations (Vineyard 2 and Vineyard 8) share a high degree of genetic material, and suggestings a high level of breeding between the subpopulations. F_IS_ was calculated to be 0.74 ± 0.04, indicating a considerable degree of inbreeding. This information has been added to Lines 784-791.*
- *We have also used GenAlEx to compare the observed heterozygosity rate, and found it to be 2 to 16 times lower than the unbiased expected heterozygosity rate for each locus, suggesting that this population has a high selfing rate (added to Lines 803-807).*

Line 703-707 : Some admixing events is far from a panmictic population. And indeed as for *S. cerevisiae*, *S. uvarum* has been described as an inbred species. The extent of admixture should be evaluated. See comment above about the interest of methods inferring admixed populations. The addition of a figure presenting this population structure may also explain uneven clustering of strains in the tree according to clusters.

The tree does not contain bootstrap value, which may also explain this pattern. The robustness of these nodes could be evaluated by a jacknife or a bootstrap procedure.

- *We have added an analysis of population structure of the strains of significance as compared to international strains, using InStruct software and visualized with DISTRUCT plots. Related discussions can be viewed on Lines 808-848, and the plot can be seen in Fig 5.*
- *We have also removed any statements regarding admixed or panmictic populations in reference to the tree, and have added bootstrap values above 0.5 to Fig 6.*

**Reviewer #2**

This is an interesting study looking at microbial diversity in Canadian wine systems. The main issue with the study as it stands is that appropriate analyse have not been conducted on the data and thus some conclusions are not supported. Specifically, the authors make the following statements in the conclusion section that are not supported by analyses:

Differences in fungal community composition were observed, with *H. osmophila* representing a significant proportion of the fungal community in the fermentations from one vineyard, but not the other.

>no significance is shown

- *We have completely re-written the Statistical Analysis section, and have added statistical analyses of the fungal communities and S. uvarum populations in this study, described on Lines 337-429. A discussion of statistical results has been added throughout the Results and Discussion section (Lines 496-499; 525-527; 595-598; 663-664; 729-732; and Tables 3, 4, and 5).*
- *Data analyses conducted in the revised manuscript include: (1) statistical comparisons of chemical parameters, alpha diversity (Simpson’s and Shannon’s Indices), and beta diversity (composition, based on Bray-Curtis dissimilarity); (2) rarefaction curves of species/strain richness; (3) S. uvarum population structure using InStruct; (4) unrooted tree of S. uvarum strains using k-means clustering with added bootstrap values; (5) estimations of selfing, (in)breeding, and probability of identity within the S. uvarum population.*
- *We have removed the term ‘significant’ when discussing the proportion of H. osmophila in the conclusion. However, we do not believe statistics are necessary in order to discuss the differential abundance of this species in the two vineyard treatments, which can be visually distinguished in Fig 1, and which is a small point of discussion.*

The presence of persistent non-commercial strains, as well as the phylogenetic tree generated to compare Okanagan and international strains, provides evidence for an indigenous winery-resident *S. uvarum* population in the Okanagan Valley of Canada

>no evidence for indigenous winery-resident *S. uvarum* population is provided.

- *We agree that we do not have conclusive proof for an indigenous winery-resident population. However, we did detect an overlap of 66 strains in Chardonnay fermentations at the same winery two years apart. This overlap is much higher than has been detected from studies analyzing vineyard yeast populations from successive vintages. Therefore, we suspect that the majority of strains identified in this study reside in the winery, but the non-overlapping strains (especially those with differential abundance in the fermentations from one vineyard over another) could be derived from the vineyard. We have softened the language of the Abstract and Conclusion sections (Lines 40; 956-957) and added a discussion of winery- versus vineyard-resident yeasts, taking into account both our data and previous research on this topic (Lines 618-628; 687-690; 717-720).*

The study deserves publication after these, and other less major issues listed below, are dealt with.

Other comments

Line 53-54 “aggressively competitive towards indigenous yeasts” – a couple of points here: 1) I think it is the massive inoculation size of commercial yeast that allows them to outcompete indigenous yeasts – not some other inherent advantage; 2) ‘competitive towards’ is not grammatically correct ‘more competitive than..’ would work.

- *We have changed the wording of this statement (Line 53). However, previous research supports the idea that commercial yeast strains used previously at a winery may out-compete indigenous strains, even when they are not inoculated. This discussion has been added to Lines 54-56.*

The authors cite RStudio for analyses – I appreciate they used this (as do I) but the R platform was used for analyses – Rstudio is just an interface to R.

- *We have amended this wording (Lines 225; 338).*

How many tanks were each vineyard put into? Just one? If so it isn’t really appropriate to use the sub-samples from this mixed tank as replicates – these are pseudo samples – one would need to sample multiple tanks. Either way, given the low sample numbers and non-normality of the chemical data, isn’t it more conservative and thus appropriate to use a Kruskal-Wallis text rather than an ANOVA?

- *Must from each vineyard was first transferred temporarily to a large stainless steel settling tank, before being transferred to stainless steel barrels. Three stainless steel barrels per vineyard were analyzed in this study, and the cold settling samples for microbial and chemical analysis were all taken from the stainless steel barrels. We believe our use of ‘cold settling tank’ has resulted in confusion, and have changed the name of the tank in the manuscript text to simply a ‘settling tank’ (Lines 156; 158; 159). In this study, ‘cold settling’ refers to the period of time before alcoholic fermentation started.*
- *We have chosen not to perform non-parametric tests such as Kruskal-Wallis tests, and instead to keep the parametric ANOVA tests, because our data met the assumption of homogeneity of variance, and ANOVA are considered robust to deviations from normality. We have explained and cited our reasoning on Lines 342-344.*

I think we need clarification of whether any YAN or sulphur additions were made to the juices and if so what these were.

- *This information is present on Lines 169-173.*

The logic to not perform inferential statistics on the fungal community data – due to different times of harvest and fermentation as well as location is not sound. The statistical tests are the only way to test if these communities differ – if they do one cannot determine whether location or time (or both) drove this difference – but this does not mean this should not be tested… Both between tank and within tank statistical analyses of fungal communities are absolutely required to support any statements of difference and change. Phrases such as ‘very different’ are not valid unless the are supported by an analyses. This is the core of science – not subjective interpretation but by means of objective analyses. Such analysis needs to be applied to the whole of the ‘Fugal communities’ section on the results. If not, this entire section is simply an observation and no conclusions may be drawn or made.

- *We have completely re-written the Statistical Analysis section, and have added statistical analyses of the fungal communities and S. uvarum populations in this study, described on Lines 337-429. A discussion of statistical results has been added throughout the Results and Discussion section (Lines 496-499; 525-527; 595-598; 663-664; 729-732; and Tables 3, 4, and 5).*
- *Data analyses conducted in the revised manuscript include: (1) statistical comparisons of chemical parameters, alpha diversity (Simpson’s and Shannon’s Indices), and beta diversity (composition, based on Bray-Curtis dissimilarity); (2) rarefaction curves of species/strain richness; (3) S. uvarum population structure using InStruct; (4) unrooted tree of S. uvarum strains using k-means clustering with added bootstrap values; (5) estimations of selfing, (in)breeding, and probability of identity within the S. uvarum population.*

Some basic presentation of the fungal community data is desirable: how many reads and ASVs (species) were there? What classes, orders etc. did these encapsulate?

- *We have added a basic presentation of the fungal community data on Lines 517-524. Our fungal community data were rarefied to 20,000 sequences per sample and presented as relative abundance.*

“*S. uvarum*, which dominated the fermentations from both vineyards, is a glucophilic

yeast [29], and the residual fructose observed in the late stage of fermentation suggests that this yeast is less adept at fermenting fructose than *S. cerevisiae*. This result is supported by previous research [55]” and other discussion about yeasts in lines 410-413 - the authors have not yet shown what species dominated or are present the ferments they analysed – it would be better to move these statements until after they have.

- *We have moved these statements to another section (moved to Lines 636-639).*

‘two of the dominant strains were previously identified as dominant strains

39 in uninoculated Chardonnay fermentations at the same winery two years earlier,

40 providing evidence for a winery-resident population of indigenous S. uvarum’

and

Line 590 “The reoccurrence of ‘2015 Strain 2’ and ‘2015 Strain 3’ suggests that these strains have established themselves as persistent winery residents, capable of entering and dominating fermentations in multiple vintages.”

I don’t see why this conclusion is drawn and what support it? Why is this conclusion draw over the other option – that these strains reside in the vineyards the fruit derived from – that is the reason they reoccur… there is good evidence that a large genetic variability of *Saccharomyces* yeasts reside in various vineyard habitats (soil, bark) and that these are regionally distinct populations – what direct evidence is there that live *Saccharomyces* overwinter in wineries?

- *As described above, we based our conclusions on previous research showing very little overlap between vineyard S. cerevisiae populations from vintage to vintage (Martiniuk et al. 2016; DOI:* *10.1371/journal.pone.0160259). We have softened the language of the Abstract and Conclusion sections (Lines 40; 956-957) and added a discussion of winery- versus vineyard-resident yeasts, taking into account both our data and previous research on this topic (Lines 618-628; 687-690; 717-720).*
- *There is a body of evidence that Saccharomyces and other yeasts overwinter in wineries (discussion added on Lines 625-628), and the presence of certain dominant yeast strains in fermentations from vineyards that are separated by more than 30 km (and did not enter the winery at the same time) suggests that many of these yeast strains are winery-residents. However, it is possible that some of these yeasts may live in the vineyard (and it is of course likely that this population originated in a nearby natural environment), and we have added statements to this effect (Lines 618-620; 717-720.*

Since yeast are sexual (i.e. loci recombine – the authors show heterozygosity in their data) is there a reason the authors did not analyse the microsatellite data with a method that accounts for this – some network analyses for example? The comparison of individual stains is not that powerful or appropriate – it is the degree to which the populations are genetically related and structured by analysing the alleles they share that is the better approach. Why was a ‘Structure’ analyses or similar not conducted?

- *We have added an analysis of population structure of the strains of significance as compared to international strains, using InStruct software and visualized with DISTRUCT plots. Related discussions can be viewed on Lines ##-##, and the plot can be seen in Fig 5.*

Why is a Bruvo distance of 0.3 ‘genetically similar’ – what does this mean? Can the authors quantify this in some way?

- *Bruvo distance is calculated using an algorithm that takes into account stepwise mutations, making it appropriate for use with microsatellite data. This distance is calculated on experimental data and allows the user to collapse MLGs with slight differences in allele size into a single strain category, based on similarity at a threshold value from 0 to 1. An applied threshold of 0 results in every unique MLG being classified as a different strain, and an applied threshold of 1 results in all the MLGs in a dataset being collapsed into a single strain. This information has been added to the manuscript on Lines 218-224.*
